# Intra-Individual Comparison of Physiologic [^68^Ga]Ga-PSMA-11 and [^18^F]PSMA-1007 Uptake in Ganglia in Patients with Prostate Cancer: A Retrospective, Monocentric Analysis

**DOI:** 10.3390/cancers15102787

**Published:** 2023-05-17

**Authors:** Emil Novruzov, Dominik Schmitt, Katalin Mattes-György, Markus Beu, Yuriko Mori, Mardjan Dabir, Jan Philipp Radtke, Günter Niegisch, Peter Albers, Lars Schimmöller, Gerald Antoch, Christina Antke, Frederik L. Giesel, Eduards Mamlins

**Affiliations:** 1 Department of Nuclear Medicine, Medical Faculty and University Hospital Duesseldorf, Heinrich-Heine-University Duesseldorf, 40225 Düsseldorf, Germany; 2 Department of Urology, Medical Faculty and University Hospital Duesseldorf, Heinrich-Heine-University Duesseldorf, 40225 Düsseldorf, Germany; 3 Department of Diagnostic and Interventional Radiology, Medical Faculty and University Hospital Duesseldorf, Heinrich-Heine-University Duesseldorf, 40225 Düsseldorf, Germany

**Keywords:** PSMA uptake, ganglion, ganglia, [^18^F]PSMA-1007, [^68^Ga]Ga-PSMA-11, PET

## Abstract

**Simple Summary:**

Since the introduction of PSMA imaging around a decade ago, the faint tracer uptake of ganglia in the neck, abdomen, and presacral region has been an imaging challenge due to difficult discrimination from suspicious lymph nodes. Moreover, PSMA tracers labelled with different radionuclides demonstrate varying levels of ganglion uptake despite having the same target structure on the cell membrane, resulting in distinct imaging pitfall patterns. Our study aims to investigate the underlying mechanisms of the varying detectability of PSMA ligands labelled with different radionuclides.

**Abstract:**

Background: Several studies indicate, particularly in the case of [18F]PSMA-1007, a relatively high rate of detection of ganglia in PSMA PET imaging. Ganglia are an integral part of the sympathetic portion of the autonomous nervous system. To date, no studies have directly compared [68Ga]Ga-PSMA-11 and [18F]PSMA-1007 ganglionic uptake intra-individually and analyzed the underlying molecular and physical mechanisms of different detection rates. With this monocentric retrospective study, we sought to evaluate the intra-individual physiological ganglion uptake of these different PSMA ligands in evidence-based imaging for prostate cancer. Methods: Our cohort consists of 19 male patients (median age 72 ± 9 with a range of 56–85) with biochemical recurrence of prostate cancer who underwent both [68Ga]Ga-PSMA-11 and [18F]PSMA-1007 PET/CT in our clinic on the same scanner per standard care between March 2015 and March 2022. Tracer uptake was quantified according to maximum standardized uptake value (SUV_max_) for both [68Ga]Ga-PSMA-11 and [18F]PSMA-1007 PET/CT scans. Ganglia-to-background ratios (GBRs) were determined to quantify the image contrast through dividing the SUV_max_ of the ganglia by the background value (SUV_max_ of blood pool in the descending aorta, fatty tissue, and skeletal muscle in gluteal region). We used descriptive analyses for demographics and tumor characteristics and performed two-way repeated-measures ANOVA (analysis of variance) for SUV metrics including GBR measurements. Results: In total, we examined 101 ganglia with [^18^F]PSMA-1007 scanning, localized mostly in pairs as stellate, coeliac, and sacral, of which 76 were also detected with [^68^Ga]Ga-PSMA-11 PET/CT scanning. There was no statistically significant difference in PSMA uptake in terms of SUV_max_ between [^18^F]PSMA-1007 and [^68^Ga]Ga-PSMA-11 (*p* value: 0.052). In contrast, the comparison of GBRs revealed a higher detectability rate of ganglia with [^18^F]PSMA-1007 imaging (*p* < 0.001). Furthermore, a separate comparison of ganglia with respect to their anatomical location also demonstrated statistically significant differences both within and between [^18^F]PSMA-1007 and [^68^Ga]Ga-PSMA-11 PET/CT scans. Conclusion: Given the impression of more accentuated [^18^F]PSMA-1007 uptake in ganglia compared with ^68^Ga-labelled counterparts, our study demonstrated that the better detectability of ganglia is not due to more intense [^18^F]PSMA-1007 uptake by these small structures but to much more favorable physical properties of the radionuclide ^18^F. The most relevant limitations of our study are its retrospective design and the small patient cohort.

## 1. Introduction

Prostate cancer is the second leading malignancy of the male population worldwide and the most frequent cancer in elderly men (>60 years) with high morbidity and mortality, especially in advanced cancer stages [1]. The correct diagnosis, treatment, and follow-up of prostate cancer patients have been very challenging due to the insidious onset and progression of the tumor and a very high rate of postoperative recurrences (27–53%). The accurate detection of culprit lesion(s) in biochemical recurrence (BCR), or the setting of post-therapeutic relapse with elevated tumor marker levels (prostate-specific antigen, PSA), represents the most challenging step in prostate cancer management, which regularly escapes conventional imaging modalities such as computed tomography (CT), magnetic resonance imaging (MRI), or bone scintigraphy. This unmet clinical need has directed attention to molecular diagnostics with prostate-specific membrane antigen (PSMA) imaging [2,3]. 

The introduction of [^68^Ga]Ga-PSMA-11 marks a revolutionary milestone in prostate cancer management and currently represents the most widely used PSMA PET tracer worldwide. The alternative ^68^Ga-labelled PSMA tracer, [^68^Ga]Ga-PSMA-617, offers a suitable substrate for theranostic applications, whereas diagnostic use is not as practical as [^68^Ga]Ga-PSMA-11 due to slower tumor accumulation and clearance kinetics. However, Ga labelling exhibits several technical and practical disadvantages such as the need for generator-based production with a short half-life, which significantly limits the supply of off-center nuclear medicine facilities and, thus, high-volume use as well as economic benefit. In fact, commercially available ^68^Ge/^68^Ga generators can offer only a maximum activity of 1.85 GBq of ^68^Ga (88.9% β+; half-life: 67.71 min). In addition, ^68^Ga has a high positron energy with a wide positron range (1899.1 keV, mean 890 keV), leading to limitations of spatial resolution and, thus, indirectly, also to more partial-volume effect than ^18^F-labelled tracers. A number of ^18^F-labelled PSMA-targeting radiotracers (96.7% β+; half-life: 109.77 min; 633.5 keV, mean keV 250) have been developed to overcome the aforementioned drawbacks of Ga labelling, of which [^18^F]PSMA-1007 has been considered to be the most significant PSMA PET tracer due to very favorable biodistribution with a predominantly hepatobiliary clearance, also enabling large-scale, cost-effective, off-center use due to cyclotron production. Moreover, a low energy profile with a shorter positron range allows a better spatial resolution than ^68^Ga (Table 1) [4,5,6,7]. 

Several studies indicate, particularly in the case of [^18^F]PSMA-1007, a relatively high rate of detection of benign findings in PSMA PET imaging, ganglia being the most common [8,9]. Ganglia are an integral part of the sympathetic portion of the autonomous nervous system. From the upper neck to the coccyx, sympathetic chain ganglia are found bilaterally along the entire length of the vertebral column. The accurate discrimination of ganglia from malignant lesions as well as various detectability rates of ganglia with different PSMA ligands have been considered as some of the major pitfall sources along non-specific bone lesions in clinical practice [8,9,10,11,12]. 

To date, no studies have directly compared [^68^Ga]Ga-PSMA-11 and [^18^F]PSMA-1007 ganglionic uptake intra-individually and analyzed the underlying molecular and physical mechanisms of different detection rates. With this monocentric retrospective study, we sought to evaluate the intra-individual physiological ganglion uptake of these PSMA ligands in evidence-based imaging for prostate cancer.

## 2. Materials and Methods

### 2.1. Patient Population

Nineteen male patients (median age 72 ± 9 with a range of 56–85) with biochemical recurrence of prostate cancer were included in this study, who underwent both [^68^Ga]Ga-PSMA-11 and [^18^F]PSMA-1007 PET/CT in our clinic per standard care in follow-up between March 2015 and March 2022. The data were anonymized and retrospectively analyzed. The study received approval from the Ethical Committee of the Medical Faculty of Heinrich-Heine-University Duesseldorf, Germany (Study-Nr.: 2022-2070).

### 2.2. PET Image Acquisition

Imaging data were acquired 72 (median, range of 56–130) minutes after intravenous application of [^68^Ga]Ga-PSMA-11 (injected median activity 150 MBq, range of 120–185) and 123 min (median, range of 98–138) after intravenous administration of [^18^F]PSMA-1007 (injected median activity 235 MBq, range of 209–278), respectively, with subsequent whole-body imaging. All PET scans were acquired in 3D mode with a body-weight-adjusted acquisition time of 3–4 min/bed position on the same PET/CT scanner with Siemens Biograph 128 mCT (Supplementary Table S1). All patients were monitored regarding any new symptoms or abnormalities up to 30 min after the end of the examination. The median time interval between the scans was 34 months (range of 9–62 months).

### 2.3. Image Analysis

Tracer uptake was quantified according to maximum standardized uptake value (SUV_max_) for both [^68^Ga]Ga-PSMA-11 and [^18^F]PSMA-1007 PET/CT scans. Circular regions of interest (ROIs) were placed on axial slices around ganglia with focally increased tracer uptake (by EN with 5 years of imaging experience; supervised by EM with 10 years of imaging experience and FLG with more than 20 years of imaging experience) and were automatically incorporated into a three-dimensional volume of interest with a 40% iso-contouring approach using Syngo.via software (ESoft; Siemens Healthineers, Erlangen, Germany). SUVs were determined via drawing volumes of interest (VOIs) on ganglia, which were then correlated anatomically with CT images for discrimination from lymph node metastases. Ganglia were defined as such: if the focal or tear-drop- or ribbon-shaped anatomical structures are found in typical paravertebral sites with tracer uptake. Ganglion-to-background ratios (GBRs) were determined to quantify the image contrast through dividing the SUV_max_ of the ganglia by the background value (SUV_max_ of blood pool in the descending aorta, fatty tissue, and skeletal muscle in gluteal region). Finally, we conducted an intra-individual correlation of the detected ganglia (single or pairwise stellate, coeliac, and presacral ganglia) with increased tracer uptake for the determination of further comparability.

### 2.4. Statistical Analysis

We used descriptive analyses for demographics and tumor characteristics and performed two-way repeated-measures ANOVA (analysis of variance) for SUV metrics including GBR measurements that are defined as quantitative variables, whereas the sites of the ganglia and the tracer were considered as categorical variables. These statistical analyses were performed with SigmaStat version 3.5 (Systat Software, Inc., San Jose, CA, USA) and Microsoft Excel (Microsoft Corporation, Redmond, DC, USA). A *p* value of <0.05 was considered statistically significant.

## 3. Results

In total, we identified 101 ganglia in a cohort of 19 male patients with [^18^F]PSMA-1007 scanning, localized mostly in pairs as stellate, coeliac, and presacral, of which 76 were also detected using [^68^Ga]Ga-PSMA-11 PET/CT scanning (Table 2). There was no statistically significant difference in PSMA uptake in terms of SUV_max_ between [^68^Ga]Ga-PSMA-11 and [^18^F]PSMA-1007 PET/CT (*p* value: 0.052). In this regard, a separate comparison of PSMA tracers among anatomical sites of detected ganglia also revealed no statistically significant difference in terms of SUV_max_ (*p* value: 0.238). Nevertheless, coeliac ganglia exhibited the highest uptake, followed by stellate and presacral ganglia for both tracers (Table 3).

In contrast, a comparison of GBRs with respect to blood pool, fatty tissue, and skeletal muscle revealed a significantly higher uptake of [^18^F]PSMA-1007 (*p* < 0.001), i.e., sharper imaging contrast. Furthermore, a separate comparison of ganglia in terms of GBR with respect to their anatomical location also demonstrated statistically significant differences between and also within [^68^Ga]Ga-PSMA-11 and [^18^F]PSMA-1007 PET/CT scans (Table 4). 

## 4. Discussion

After the implementation and widespread use of [^18^F]PSMA-1007 in prostate cancer management, clinicians have been reporting a high number of benign, non-prostatic structures, in particular ganglia, in comparison to the current most widely used PSMA PET tracer [^68^Ga]Ga-PSMA-11. Of the 22–23 pairs of ganglia that comprise the sympathetic chain, the stellate ganglia (which emerged from the combination of C7 and T1 ganglia during evolution), the coeliac ganglia (at the level of T11/12), and the sacral ganglia have been predominantly shown to exhibit a substantial PSMA uptake. Despite multiplex, clinical, and imaging correlation methods, it has occasionally led to pitfalls and misdiagnoses in regular clinical care [9,12,13]. 

**Figure 1:**
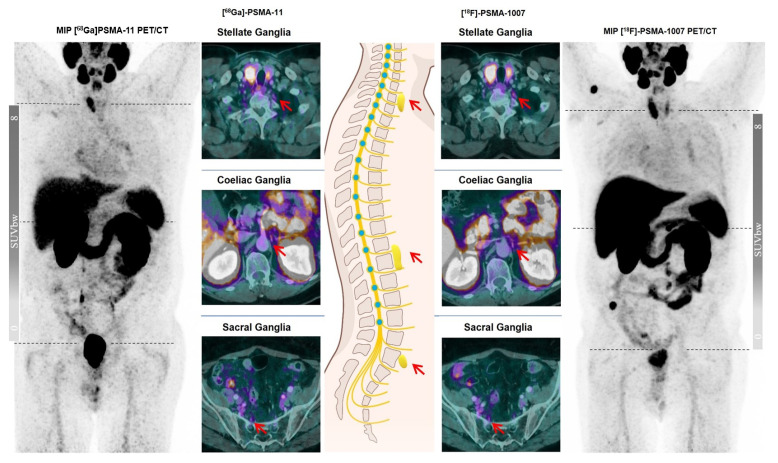
A 79-year-old male patient in follow-up after biochemical recurrence of prostate cancer with varying visual detectability of ganglia at the typical sites (red arrows) on different PSMA ligands.

With this monocentric, retrospective study, we aimed to evaluate the underlying molecular and physical mechanisms of varying tracer uptake of the ganglia via comparing the two widely used PSMA PET tracers [^68^Ga]Ga-PSMA-11 and [^18^F]PSMA-1007 on an intra-individual basis using the same PET/CT scanner. We observed a slightly less intense [^68^Ga]Ga-PSMA-11 uptake of the ganglia in terms of SUV_max_ and also a smaller number of detected ganglia than [^18^F]PSMA-1007, even though the statistical assessment revealed no significant difference (Figure 1). Given the anatomical location of the ganglia, coeliac ganglia exhibited the most intense tracer uptake followed by stellate and then presacral ganglia for both tracers. However, the comparison of PSMA uptake in ganglia in terms of GBR showed an unequivocal statistical superiority of [^18^F]PSMA-1007 over [^68^Ga]Ga-PSMA-11 across all anatomical locations. 

This is a very noteworthy finding, as the reader might detect a greater number of ganglia in [^18^F]PSMA-1007 PET imaging not necessarily because of more intense tracer uptake than [^68^Ga]Ga-PSMA-11, but because of much better GBR. As Giesel et al. reported, [^18^F]PSMA-1007 exhibits slightly lower tracer kinetics than [^68^Ga]Ga-PSMA-11, so that favorable GBR for [^18^F]PSMA-1007 might only be explained by the distinct physical characteristics of different radionuclides [14]. 

Image quality or spatial resolution is a result of multiple vectors such as detector-element geometry, annihilation acollinearity, and positron range. In a study with the same state-of-the-art digital PET/CT scanners, the positron range of different tracers is the most important determining factor for tracer-related differences in spatial resolution and even quality of activity quantitation. Positrons arising from the decay of ^68^Ga feature an endpoint energy three times higher than those of ^18^F and therefore have a much greater mean range in tissue (Table 1). Furthermore, given the shorter half-life and lower positron yield of ^68^Ga than ^18^F (67.7 min vs. 109.7 min and 89.14% vs. 96.86%, respectively), there is higher activity of ^18^F in tissue despite adjusted acquisition times for the different tracer. This causes greater positron flux, resolution, better count statistics, and, thus, greater lesion detectability with [^18^F]PSMA-1007. In particular, the image quality and spatial resolution of lesions smaller than 13 mm in diameter are shown to be impaired by radionuclides with higher endpoint energy of positrons (e.g., ^68^Ga). Besides, this also affects the quantitative image parameters between lesions of different sizes, as the so-called partial volume effect worsens the detection sensitivity [4,15]. 

The relevant limitations of this study are its retrospective design and small patient cohort. Furthermore, no histopathological correlation was performed, but since the ganglia do not represent unequivocal findings, histological confirmation is not applicable. The time interval between PET scans was also not considered to be a relevant factor for fluctuating tracer uptake, as the study groups of olde Heuvel et al. and Mamlins et al. demonstrated largely stable [^68^Ga]Ga-PSMA-11 and [^18^F]PSMA-1007 uptake of ganglia, respectively, over a long period of time [11,16]. Moreover, the investigation by Alberts et al. showed no reliable discrimination between ganglia and malignant lesions with respect to tracer uptake kinetics in late images, which makes the acquisition of delayed images redundant [8]. In addition, as all patients underwent both examinations on the same PET/CT scanner with the same reconstruction algorithms and parameters, no procedural bias is to be expected. 

## 5. Conclusions

To our best knowledge, this is the first investigation to evaluate the physiologic [^68^Ga]Ga-PSMA-11 and [^18^F]PSMA-1007 uptake in ganglia in patients with prostate cancer with respect to its underlying molecular and physical mechanisms on an intra-individual basis using the same PET/CT scanner in a monocentric, retrospective design. Given the impression of greater [^18^F]PSMA-1007 uptake in ganglia, our study demonstrated that the better detectability of small lesions, particularly ganglia, is not due to more intense [^18^F]PSMA-1007 uptake by these structures, but to better ganglion-to-background ratio (GBR) due to the much more favorable physical properties of the radionuclide ^18^F. In summary, the different ganglion detectability demonstrated by different PSMA tracers appears to be based on the physical properties of the distinct radionuclides.

## Figures and Tables

**Table 1:** Differences in mean positron range between ^18^F and ^68^Ga in various human tissues (adapted from [4]).

	FLUORINE-18	GALLIUM-68
	Mean positron range (mm)	Mean positron range (mm)
BONE TISSUE	0.19	0.67
SOFT TISSUE	0.27	1.05
ADIPOSE TISSUE	0.33	1.17
LUNG TISSUE	0.80	3.32

**Table 2:** Number of detected ganglia using [^68^Ga]Ga-PSMA-11 and [^18^F]PSMA-1007 PET/CT scanning.

Number of Detected Ganglia	[^68^Ga]Ga-PSMA-11	[^18^F]PSMA-1007
Stellate	32	38
Coeliac	24	38
Presacral	20	25
Total	76	101

**Table 3:** Tracer uptake in ganglia in terms of SUV_max_ (a) and comparison of tracer uptake within anatomical locations (b).

(a)		
Tracer Uptake (in SUV_max_)	[^68^Ga]Ga-PSMA-11	[^18^F]PSMA-1007
Stellate (mean ± SD)	2.30 (±0.16)	2.91 (±0.16)
Coeliac (mean ± SD)	2.91 (±0.16)	3.18 (±0.15)
Presacral (mean ± SD)	1.73 (±0.21)	1.73 (±0.19)
(b)		
Comparison (using Holm–Sidak method)	[^68^Ga]Ga-PSMA-11 and [^18^F]PSMA-1007 uptake	
Coeliac vs. Stellate (diff. of means)	0.44 (*p* < 0.001)	
Stellate vs. Presacral (diff. of means)	0.87 (*p* < 0.001)	
Coeliac vs. Presacral (diff. of means)	1.31 (*p* = 0.011)	

**Table 4:** Tracer uptake in ganglia in terms of GBR and comparison of tracer uptake within anatomical locations with respect to blood pool (a,b), adipose tissue (c,d), and skeletal muscle (e,f).

(a)			
Tracer Uptake (mean GBR with respect to blood pool)	[^68^Ga]Ga-PSMA-11	[^18^F]PSMA-1007	*p* Value
Stellate	1.19 (±0.08)	1.97 (±0.07)	<0.001
Coeliac	1.55 (±0.09)	2.23 (±0.07)	<0.001
Presacral	0.89 (±0.10)	1.25 (±0.09)	0.011
In Total	1.21 (±0.05)	1.81 (±0.04)	<0.001
(b)			
Comparison *(GBR with respect to blood pool)*	[^68^Ga]Ga-PSMA-11	[^18^F]PSMA-1007	
Coeliac vs. Stellate (diff. of means)	0.36 (*p* = 0.01)	0.26 (*p* = 0.017)	
Stellate vs. Presacral (diff. of means)	0.30 (*p* = 0.02)	0.71 (*p* < 0.001)	
Coeliac vs. Presacral (diff. of means)	0.66 (*p* < 0.001)	0.97 (*p* < 0.001)	
(c)			
Tracer Uptake (mean GBR with respect to adipose tissue)	[^68^Ga]Ga-PSMA-11	[^18^F]PSMA-1007	*p* Value
Stellate	5.16 (±0.50)	9.60 (±0.48)	<0.001
Coeliac	6.93 (±0.60)	10.34 (±0.46)	<0.001
Presacral	3.82 (±0.64)	5.85 (±0.57)	0.020
In Total	5.30 (±0.33)	8.60 (±0.29)	<0.001
(d)			
Comparison *(GBR with respect to adipose tissue)*	[^68^Ga]Ga-PSMA-11	[^18^F]PSMA-1007	
Coeliac vs. Stellate (diff. of means)	1.76 (*p* = 0.05)	0.73 (*p* = 0.277)	
Stellate vs. Presacral (diff. of means)	1.34 (*p* = 0.103)	3.75 (*p* < 0.001)	
Coeliac vs. Presacral (diff. of means)	3.10 (*p* = 0.002)	4.48 (*p* < 0.001)	
(e)			
Tracer Uptake (mean GBR with respect to skeletal muscle)	[^68^Ga]Ga-PSMA-11	[^18^F]PSMA-1007	*p* Value
Stellate	2.75 (±0.14)	3.19 (±0.14)	*p* = 0.029
Coeliac	3.33 (±0.17)	3.54 (±0.13)	*p* = 0.335
Presacral	1.83 (±0.18)	1.97 (±0.16)	*p* = 0.06
In Total	2.64 (±0.099)	2.90 (±0.08)	*p* = 0.044
(f)			
Comparison *(GBR with respect to skeletal muscle)*	[^68^Ga]Ga-PSMA-11	[^18^F]PSMA-1007	
Coeliac vs. Stellate (diff. of means)	0.58 (*p* = 0.012)	0.35 (*p* = 0.077)	
Stellate vs. Presacral (diff. of means)	0.91 (*p* < 0.001)	1.22 (*p* < 0.001)	
Coeliac vs. Presacral (diff. of means)	1.49 (*p* < 0.001)	1.57 (*p* < 0.001)	

## Data Availability

The data used and/or analyzed during the current study are available from the corresponding author upon reasonable request.

## Supplementary Materials

The following supporting information can be downloaded at: https://www.mdpi.com/article/10.3390/cancers15102787/s1.
