# Supplementary material for: Intra-Individual Comparison of Physiologic [68Ga]Ga-PSMA-11 and [18F]PSMA-1007 Uptake in Ganglia in Patients with Prostate Cancer: A Retrospective, Monocentric Analysis
Source: Cancers (Basel). 2023 May 17;15(10):2787. doi: 10.3390/cancers15102787 (PMC10216693; doi:10.3390/cancers15102787)
Supplement: Supplementary file 1 [file cancers-15-02787-s001.zip › cancers-2291837-supplementary.pdf]

Supplementary Table S1: Acquisition parameters of the PET/CT scanner.

**Site**	**University Hospital Düsseldorf**
PET/CT scanner	Biograph 128 mCT, Siemens
Median injected activity (MBq), [^{68}Ga]Ga-PSMA-11	150 (120 – 185)
Median injected activity (MBq), [^{18}F]PSMA-1007	235 (209 – 278)
Median post injection time (min), [^{68}Ga]Ga-PSMA-11	72 (56 - 130)
Median post injection time (min), [^{18}F]PSMA-1007	123 (98 - 138)
Median time interval (in months)	34 (9 – 62)
CT reference (mAs)	190
CT peak kilovoltage (kV)	120
CT slice thickness (mm)	2
CT slice increment (mm)	2
PET reconstruction	OSEM algorithm
Iterations	4
Subsets	8

Matrix	200 x 200
Corrections	Gaussian FWHM 5.0 mm
